# Antioxidant and Antibacterial Activities of Selected Medicinal Plants from Addis Ababa against MDR-Uropathogenic Bacteria

**DOI:** 10.3390/ijms251910281

**Published:** 2024-09-24

**Authors:** Mebrahtu Eyasu, Juana Benedí, José Antonio Romero, Sagrario Martín-Aragón

**Affiliations:** 1 Department of Pharmacology, Saint Paul’s Hospital Millennium Medical College, Gulele Sub-City, Addis Ababa P.O. Box 1271, Ethiopia; meyasu@ucm.es; 2 Department of Pharmacology, Pharmacognosy & Botany, Faculty of Pharmacy, Complutense University of Madrid, Plaza de Ramón y Cajal s/n, 28040 Madrid, Spain; jbenedi@ucm.es (J.B.); josea.romero@salud.madrid.org (J.A.R.)

**Keywords:** *Thymus schimperi*, *Rhamnus prinoides*, *Justicia schimperiana*, multidrug resistance, *Escherichia coli*, *Klebsiella pneumoniae ESBL*

## Abstract

This study determined the antioxidant and antibacterial activities of *Thymus schimperi* (Ts), *Rhamnus prinoides* (Rp), and *Justicia schimperiana* (Js) from Addis Ababa against MDR-*Uropathogenic bacteria*. Accordingly, *Thymus schimperi* had the highest total phenolic (TPC), flavonoid (TFC) and proanthocyanidin content. In Ts, the GC-MS analyses predicted 14 bioactive compounds. And among these, hexanedioic acid, bis(2-ethylhexyl) ester, thymol, and o-cymen-5-ol were the most predominant compounds, respectively. Six compounds were also predicted in Rp, where hexanedioic acid, bis(2-ethylhexyl) ester, β-D-glucopyranoside, methyl, and desulphosinigrin were the predominant, respectively. Whereas in the Js extract, five bioactive compounds were predicted, with hexanedioic acid, mono (2-ethylhexyl) ester, debrisoquine, and 8,11,14-heptadecatrienoate, methyl ester being predominant compounds, respectively. The extracts’ TPC showed a strong negative correlation with the DPPH (2,2-diphenyl-1-picrylhydrazyl) assay (r = −0.999; *p* = 0.023). In addition, the TFC correlated significantly with the ABTS (2,2′-azino-di-(3-ethylbenzthiazoline sulfonic acid)) assay (r = 0.999; *p* = 0.032). *Thymus schimperi* showed the highest antibacterial activity against clinical isolates of *Escherichia coli* and *Klebsiella pneumoniae ESBL* at 1000 mg/mL, and Ts had the lowest MIC (4 mg/mL) among evaluated extracts against *E. coli* (ATCC25922). In conclusion, Ts and Rp possess higher predicted bioactive molecules, including antioxidant and antibacterial activities, which are potentially useful in treating urinary tract infections.

## 1. Introduction

The emergence of antimicrobial resistance is a major health challenge and an urgent priority for Ethiopian policymakers. Mainly, urinary tract infections (UTIs) are of great concern and common infections since previous UTIs can predispose to future infections. And, it is mostly caused by Gram-negative bacteria, namely *E. coli*, *P. aeruginosa*, and *P. pneumoniae* [1]. In this regard, medicinal plants can be used to overcome socioeconomic and health impacts caused by multidrug-resistant (MDR) bacteria, including methicillin-resistant *Staphylococcus aureus* and MDR Gram-negative bacteria such as *E. coli* and *K. pneumoniae* [2,3]. 

Traditional medicine in Ethiopia is a significant part of healthcare delivery due to its integration into local culture which makes it accessible to the majority of the population [4]. However, regulating this practice using the nationwide safety and effectiveness frameworks used for common medicine categorizations and descriptions is challenging [5,6]. 

Uncontrolled use of antibiotics certainly contributes to the emergence of MDR against many bacterial strains [7]. In spite of it, the combinations of drug–herbals have been reported to improve the efficacy of chemotherapeutic agents, with low side-effect profiles on the living tissues [8], and antibiotics, whilst having no intrinsic antibacterial activity as well as being susceptible to the bacteria that previous antibiotics were ineffective against [9]. 

Importantly, the method to tackle these infectious diseases might be establishing the therapeutic potential of the medicinal plants. To provide reliable evidence for potential therapeutics against MDR-uropathogenic bacteria from Addis Ababa, we selected three endemic Ethiopian medicinal plant species, *Thymus schimperi*, *Rhamnus prinoides*, and *Justicia schimperiana*, used by traditional healers for the treatment of various diseases, including bacterial infections. 

*Thymus schimperi*, *Rhamnus prinoides*, and *Justicia schimperiana* are Ethiopian medicinal plants that have been used for treatment of various diseases, including bacterial infections. The genus *Thymus* is one of the genera from the family *Lamiaceae*. In Ethiopia, *Thymus* is characterized by two endemic species, namely *Thymus schimperi* and *Thymus serrulatus*. Locally, they are known as ‘*Tosign*’ (in Amharic). They grow between 2200 and 4000 m altitude, are perennial herbs, woody at the base, and are 5–40 cm high [10]. Traditionally, *T. schimperi* is claimed to treat various diseases such as renal disease, gonorrhea, cough, liver disease, and hypertension [11]. 

*In Ethiopia*, Rhamnus prinoides L’Herit (*Rhamnus prinoides*) is an endemic plant to Ethiopia that grows up to 6 m high. It is locally cultivated from 1000 to 3200 m, and widely planted in gardens [12]. It is also known as ‘Gesho’ (in Amharic). It is used as a laxative and diuretic, and as a preventive for syphilis, depurative, and cholagogue. For children with tonsillitis or with tonsils removed, some macerated leaves of ‘Gesho’ are put in the mouth to relieve the pain [13]. It has been traditionally used to add flavor to the local alcoholic drinks, ‘tella’ and ‘tej’ (in Amharic), brewed from fermented barley, sorghum, or finger millet [14]. According to [12], *Rhamnus prinoides* is used to treat different kinds of bacterial infections and exhibits a strong antioxidant property due to the presence of polyphenols in sufficient amounts.

*Justicia schimperiana* is a plant belonging to the family of *Acanthaceae*. *Justicia schimperiana* has different local names in Ethiopia, including ‘dhumuugaa’ (in Afaan Oromoo), ‘Sensel’ or ‘Simiza’ (in Amharic), and ‘Surpa’, ‘Kasha’, or ‘Keteso’ (in Sidama). This plant has several medicinal uses in different areas of Ethiopia. Accordingly, ethnobotanical study reports show that the plant is used in the treatment of various ailments such as evil eye, hepatitis B (jaundice), rabies, asthma, common cold, stomachache, diarrhea, tapeworm infestation, anthrax, wound, external parasite, ascariasis, and skin irritation [15,16]. Moreover, the crude extract of *J. schimperiana* has been shown to exhibit antibacterial activity against *Neisseria gonorrhea* and *Shigella flexineri* [17]. 

These study plants were selected due to their widespread availability and because of the inhabitants’ belief in their antibacterial abilities. Moreover, their effective documented properties [12,13,15,16,17] made them suitable candidates. According to [18], due to safety concerns, these study plants are wild edible plants in Ethiopia. Furthermore, those plants, which exhibit a broad spectrum of antimicrobial effects, could possibly control the impairments associated with MDR microbes [19]. 

These studied plants have been checked with “World Flora Online” (www.worldfloraonline.org), and the names of the plants correspond to the latest revision and have accepted names. To solve MDR, scientific studies of medicinal plants are necessary to sustain the traditional potential claims of medicinal activities. Therefore, this study aimed to evaluate the quantitative and qualitative phytochemical analysis, GC-MS characterization of bioactive constituents of the plants, and in vitro antioxidant and antibacterial activities of the three extracts against MDR-uropathogenic *E. coli* and *K. pneumoniae ESBL* and the reference strains, as well as the synergistic activities between ciprofloxacin and each of these three plant extracts. Briefly, this study aimed to establish the antibacterial potential of the three aforementioned plants against MDR-uropathogenic *E. coli* and *K. pneumoniae ESBL*. 

## 2. Results

### 2.1. Qualitative Phytochemical Analysis

The findings of the preliminary phytochemical analysis (in qualitative form) showed the presence or absence of the secondary metabolites in the three hydromethanolic leave crude extracts and are described in Table 1. Accordingly, the results of the tests on *T. schimperi* showed included a maximum of six types of phytochemical constituent groups, such as alkaloids, coumarins, flavonoids, phenols, saponins, and terpenoids. Next, *R. prinoides* contained phenol, tannin, flavonoids, coumarins, anthocyanin, alkaloids, and saponins. Finally, the third plant, the *J. schimperiana* extract, contained five types of phytochemical groups, such as saponin, coumarins, flavonoids, phenol, and tannin. 

### 2.2. Quantitative Phytochemical Analysis

**Figure 1:**
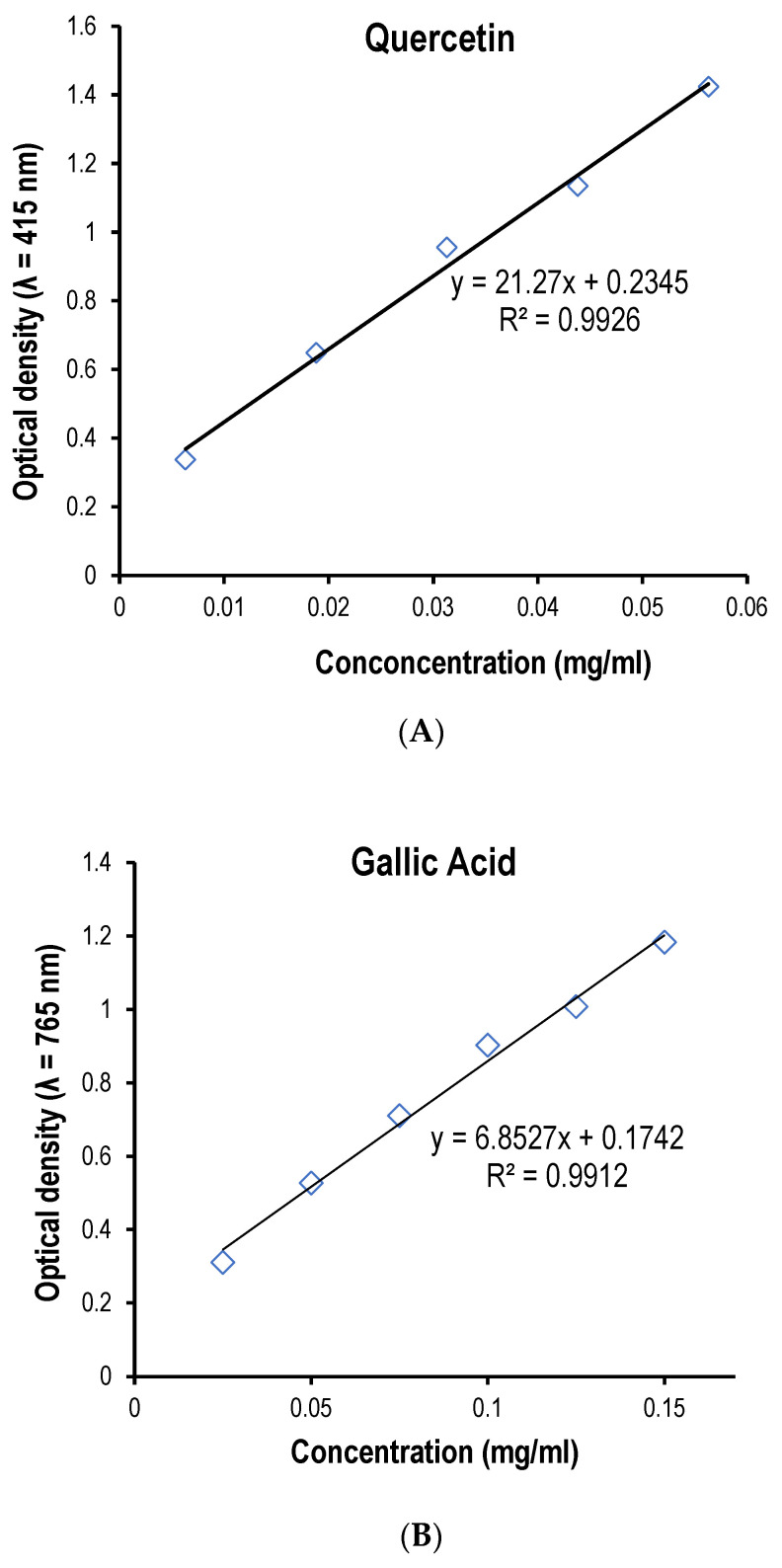

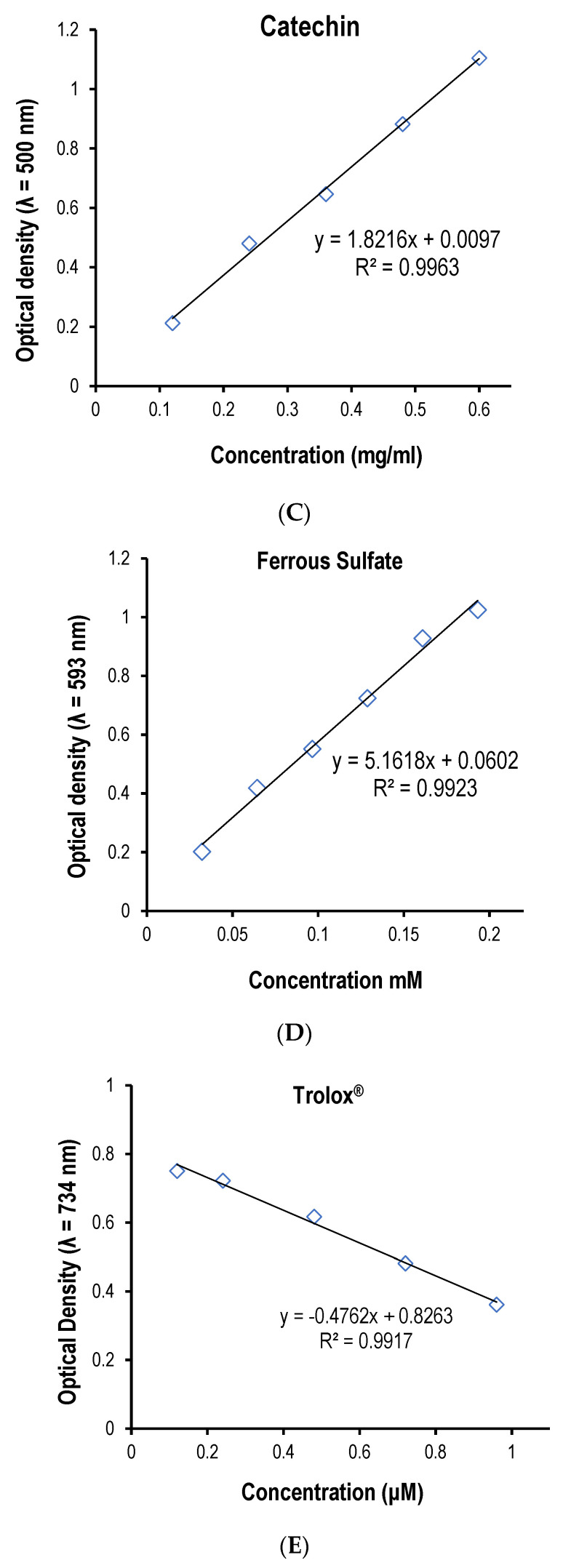
(**A**) Linear regression calibration curve of the standard quercetin for the determination of flavonoids. (**B**) Linear regression calibration curve of the standard gallic acid for the determination of the Total Phenol Content. (**C**) Linear regression calibration curve of the standard catechin for the determination of proanthocyanidins. (**D**) Linear regression calibration curve of the standard ferrous sulfate in the FRAP assay. (**E**) The linear regression curve of the standard compound (Trolox^®^) used in the ABTS assay.

This study carried out a quantitative phytochemical analysis of the three hydromethanolic crude leaf extracts of *T. schimperi, R. prinoides,* and *J. shimperiana*. The analysis aimed to determine the total phenolic content, flavonoids, and proanthocyanidins, and the findings are presented in Table 2. The results demonstrated that the total flavonoid content was 90.39 ± 0.35 Quercetin Equivalence (QE)/100 g of dry *R. prinoides* material; 82.43 ± 2.53 QE/100 g of dry *J. schimperiana* material; and 227.96 ± 3.69 QE/100 g of dry *T. schimperi* material. Among the three crude extracts, *T. schimperi* had the highest average (±SD) in phytochemical contents of flavonoids (227.96 ± 3.69), phenolics (274.50 ± 5.29), and proanthocyanidins (1618.06 ± 1.32) (Table 2). The concentration–response (calibration) curve of the standard chemical quercetin in the determination of flavonoids contents is plotted in Figure 1A. 

As shown in Table 2, the total phenolic contents of the three hydromethanolic of crude extracts were determined using the standard gallic acid. The standard gallic acid linear regression calibration curve is presented in Figure 1B. Total phenolic content of the extract was calculated as gallic acid equivalent/100 g of the dry plant materials. And, accordingly, the highest phenolic quantity was detected in *T. schimperi* (274.50 ± 5.29), followed by *R. prinoides* (229.21 ± 2.82) (Table 2). 

Among the three extracts, the highest proanthocyanidin content (catechin equivalence per 100 g of the dry plant material) was found in *T. schimperi* (1618.06 ± 1.32), followed by *J. schimperiana* (1037.18 ± 4.06) (Table 2). The linear regression curve of the catechin standard concentration (mg/mL) versus the optical density is shown in Figure 1C. 

### 2.3. GC-MS Analyses

As shown in Table 3, the results obtained from GC-MS analyses of the hydromethanolic extract of *T. schimperi* clearly revealed the identification and presence of 14 bioactive compounds. The identified and matched compounds were represented with the name of the compound, molecular formula, molecular weight, retention time, percentage composition of the area, nature of the compounds, and chemical structure. 

**Figure 2:**
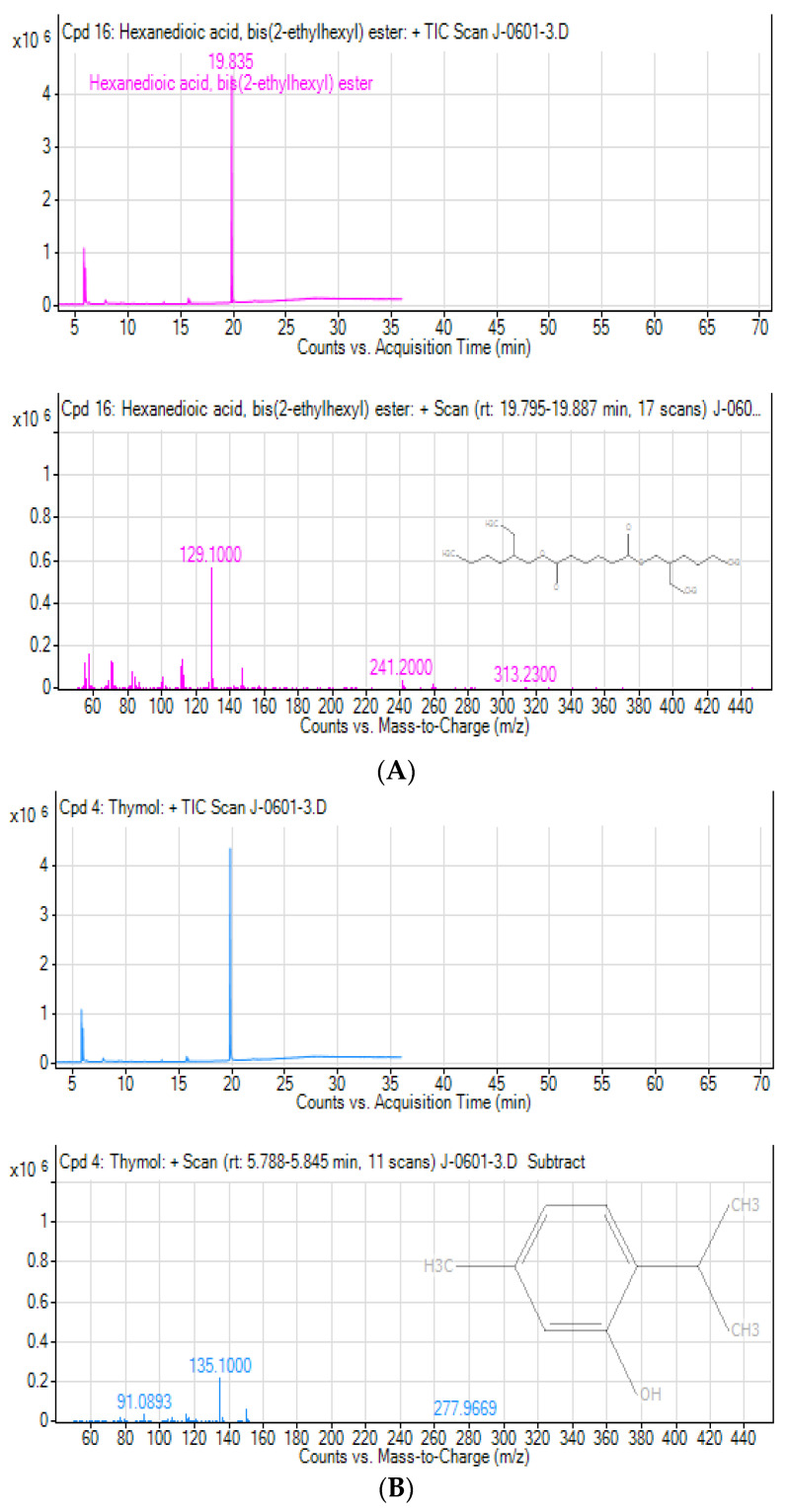

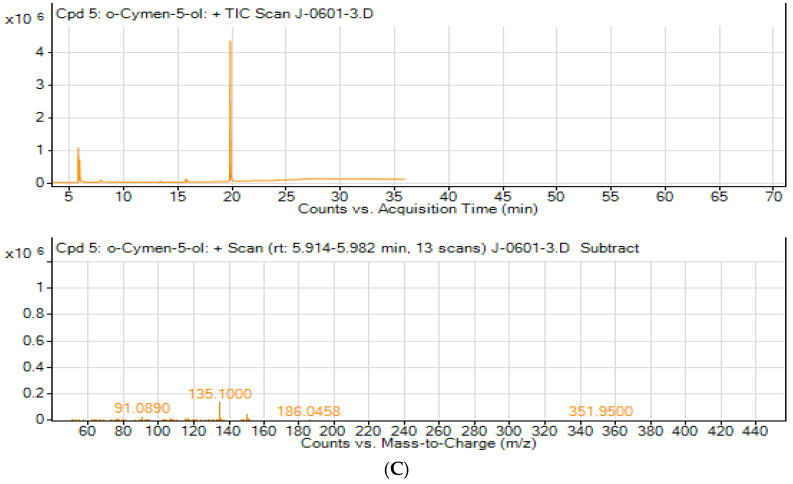
(**A**–**C**). Peak fragmentation of the chromatography mass spectrum of the three major compounds from *T. schimperi*.

**Figure 3:**
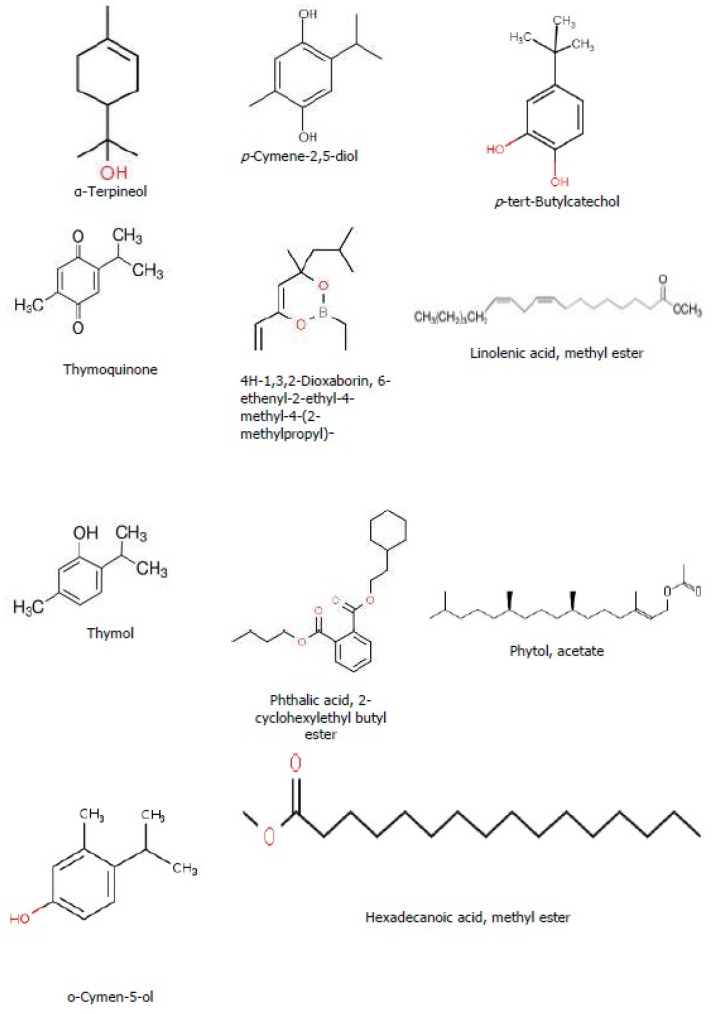

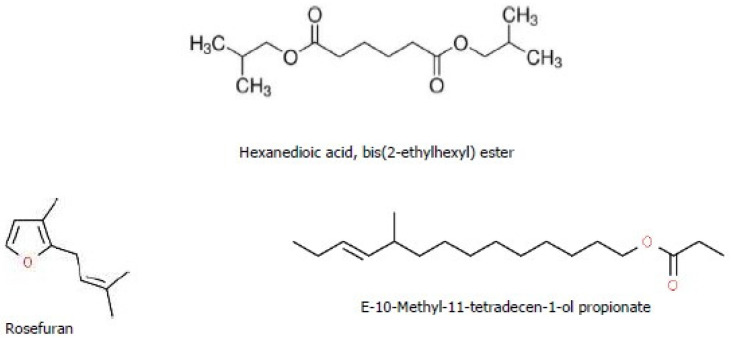
Chemical structure of the bioactive compounds identified through GC-MS analyses from the hydromethanolic *T. schimperi* crude extract.

**Figure 4:**
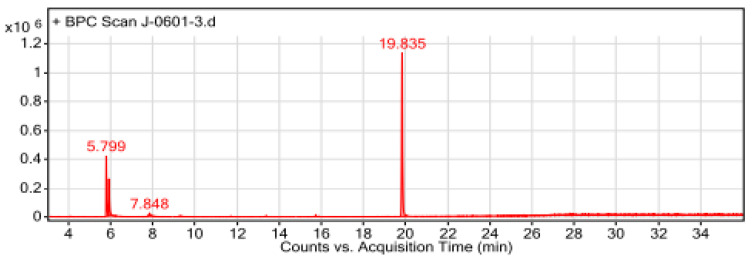
The GC-MS chromatogram of the hydromethanolic crude extract of *T. schimperi.*

During the analyses, the first compound identified (peak 1) was observed at a retention time of 4.666 min and the last compound (peak 14) was identified at the longest retention time of (19.835 min). Among the compounds eluted, the major compounds identified in this *T. schimperi* crude extract were hexanedioic acid, bis (2-ethylhexyl) ester (RT: 19.835; 73.88%); thymol (RT: 5.799; 11.68%); O-Cymen-5-ol (RT: 5.931; 7.95%); and p-tert-Butylcatechol (RT: 7.853; 2.19%), respectively, along with the other minor constituents. In the GC-MS analyses, the chromatogram shows the peak area separation of the components. The mass spectra of the three major bioactive compounds in *T. schimperi* crude extract confirmed are presented in Figure 2A–C. The chemical structure of the predicted bioactive compounds in *thymus schimperi* is presented in Figure 3. The GC-MS chromatogram of the hydromethanolic crude extract of *T. schimperi* is presented in Figure 4.

**Figure 5:**
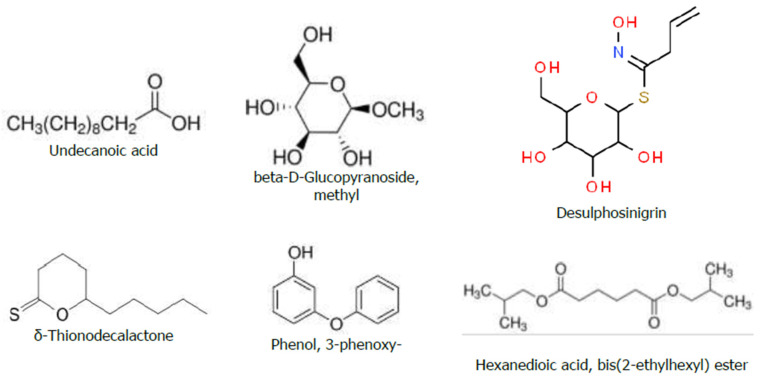
Chemical structure of the bioactive compounds identified through GC-MS analyses from the hydromethanolic *R. prinoides* crude extract.

**Figure 6:**
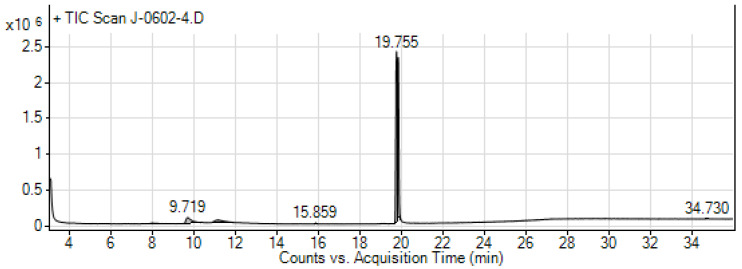
The GC-MS chromatogram of the hydromethanolic crude extract of *R. prinoides.*

In the GC-MS analyses of the hydromethanolic crude extract of *R. prinoides*, six compounds were identified. In these analyses, the three abundant bioactive compounds identified among the eluted components were the following: hexanedioic acid, bis (2-ethylhexyl) ester (RT: 19.755; 79.36%); beta-D-Glucopyranoside, methyl (RT: 9.719; 10.03%); and Desulphosinigrin (RT: 11.132; 8.28%) (Table 4). The chemical structure of the predicted bioactive compounds in *R. prinoides* is presented in Figure 5. The GC-MS chromatogram of hydromethanolic crude extract of *R. prinoides* is presented in Figure 6. 

**Figure 7:**
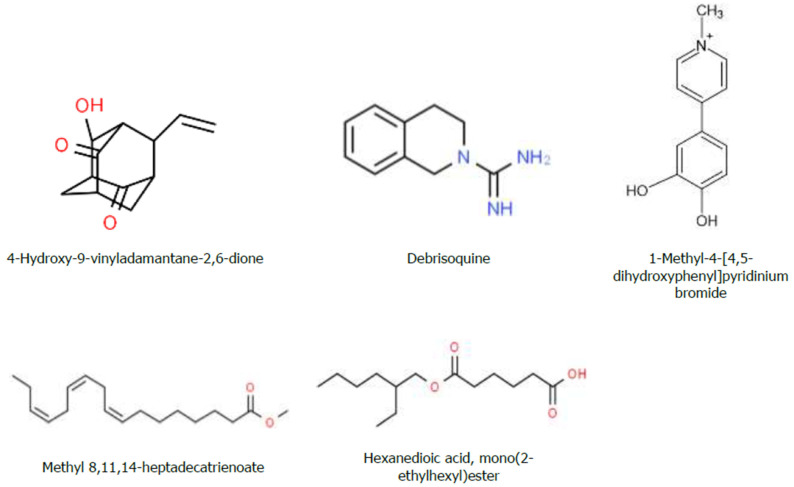
Chemical structure of the bioactive compounds identified through GC-MS analysis from the hydromethanolic crude *J. schimperiana* extract.

**Figure 8:**
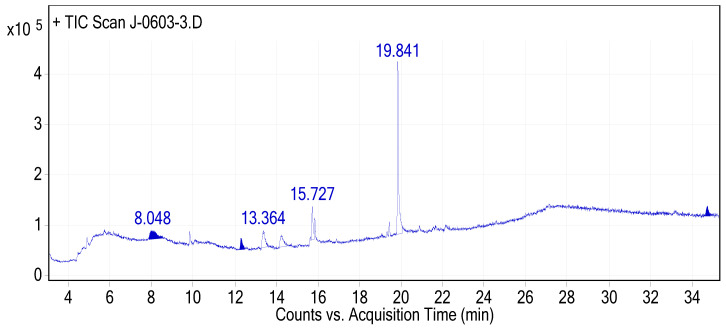
The GC-MS chromatogram of the hydromethanolic crude extract of *J. schimperiana.*

In this study, the GC-MS analyses of the hydromethanolic crude extract of *J. schimperiana* confirmed the presence of five bioactive compounds. The predominant compound was hexanedioic acid, mono (2-ethylhexyl) ester (RT: 19.841; 59.6% peak area). The second abundantly occurring bioactive compound was debrisoquine (RT: 13.364; 12.18%), followed by methyl 8, 11, 14-heptadecatrienoate (RT: 15.727; 10.79%) (Table 5). The chemical structure of the predicted bioactive compounds in *J. schimperiana* is presented in Figure 7. The GC-MS chromatogram of hydromethanolic crude extract of *J. schimperiana* is presented in Figure 8. 

### 2.4. DPPH Radical Scavenging Assay

The result of various concentrations versus the DPPH scavenging activity in terms of percentage inhibition of the three hydromethanolic extracts with the positive control, ascorbic acid, is shown in Table 6. The synthetic antioxidant ascorbic acid was used as a positive control at the same concentrations of as the tested extracts. The percentage inhibition of DPPH scavenging activities of the three hydromethanolic crude extracts was evaluated at concentrations of 0.02–0.2 mg/mL. There was an increase in DPPH radical scavenging activity with increasing concentrations of the extracts. At a 0.2 mg/mL concentration, the percentage inhibition of all the extracts was relatively higher than the rest of the concentration. The scavenging effect of the standard drug ascorbic acid was higher than all the extracts. However, in all the extracts (*T. schimperi*, *R. prinoides* and *J. schimperiana*) tested, *T. schimperi* exhibited a comparable antioxidant activity with that of standard ascorbic acid at varying concentrations tested (0.02–0.2 mg/mL) (Table 6). 

### 2.5. Ferric Reducing Antioxidant Power Assay (FRAP Assay)

**Figure 9:**
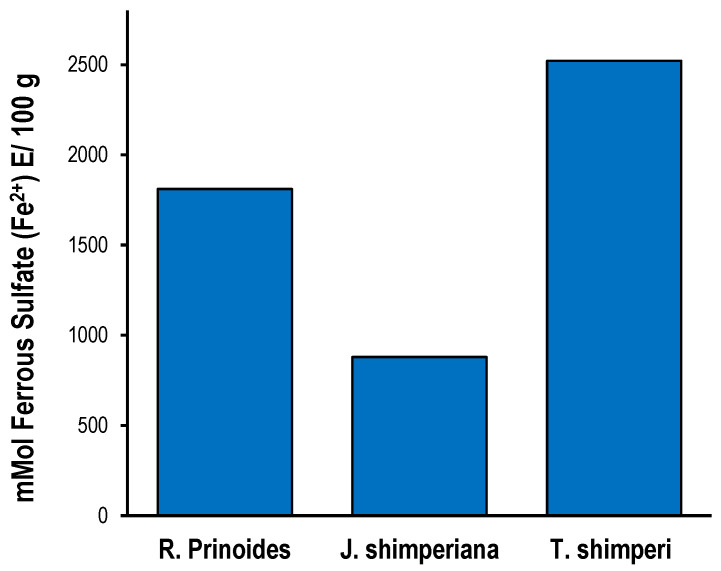
Ferric reducing antioxidant power assay (FRAP assay) of *R. prinoides*, *J. schimperiana* and *T. schimperi* of hydromethanolic leaf crude extracts. The results are expressed as mmol ferrous sulfate (Fe^2+^) equivalence/100 g. The bars represent the mean ± standard deviation (SD) (*n* = 3).

The FRAP assay results of the three hydromethanolic leave crude extracts were expressed as mmolee equivalents of ferrous sulfate and are shown in Figure 9. Similar to the DPPH assay, all the extracts showed an antioxidant activity in the FRAP assay. The *T. schimperi* extract showed the highest reducing antioxidant power among the extracts with 2521.60 mmole ferrous sulfate Eq (mmole Fe^2+^), followed by *R. prinoides* (1810.55 mmole Fe^2+^). The linear regression curve of the standard compound (ferrous sulfate) used in the FRAP assay at different concentrations is presented in Figure 1D. 

### 2.6. ABTS Assay

**Figure 10:**
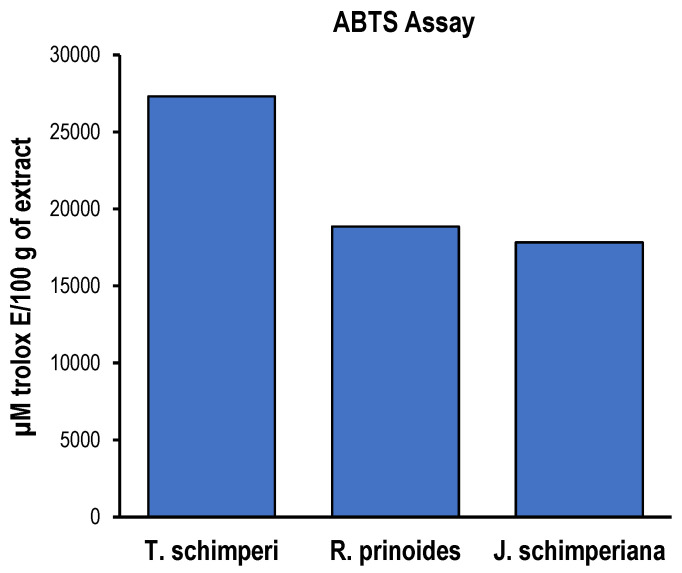
ABTS assay of *R. prinoides*, *J. schimperiana and T. Schimperi* of hydromethanolic extracts. Results are expressed as µmole *Trolox*^®^ equivalence/100 g of dried extract. Data are represented as the mean ± SD (*n* = 3).

The ABTS radical scavenging activity of the three hydromethanolic leaf extracts with the reference compound, 6-hydroxy-2,5,7,8-tetramethylchroman-2-carboxylic acid (Trolox^®^, St. Louis, MO, USA), is shown in Figure 10. The ABTS assay results were expressed as a µmole equivalent of Trolox^®^. The ABTS radical scavenging activity of the extracts was evaluated at 40 µL (n = 3; triplicate). Therefore, the findings revealed that *T. schimperi* was had the highest antioxidant activity with a mean of 27,296.65 µmole Trolox Eq (µmole Trolox^®^), followed by *R. prinoides* (18,857.62 µmole Trolox^®^). The linear regression curve of the standard compound (Trolox^®^) in the ABTS assay used at different concentrations is presented in Figure 1E. 

### 2.7. In Vitro Evaluation of Antibacterial Activity

Except for *J. schimperiana*, the rest of the results of this study showed the hydromethanolic crude leave leaf extracts and their combination had antibacterial activity against the patient-isolated multi drug multidrug-resistant (MDR) *E. coli* compared to the negative control. According to the records, *T. Schimperi* earned the highest diameter of the zone of inhibition (DZI: 20.00 ± 0.00 mm) at 1000 mg/mL concentration compared to all the uncombined tested extracts. However, the extract–extract combination of *T. Schimperi* and *R. Prinoides* had the same antibacterial activity as uncombined the *T. Schimperi* extract at the maximum tested concentration (1000 mg/mL) (Table 7). 

Largely, all the tested extracts except *J. schimperiana* showed a concentration dependent increase in antibacterial activity in the evaluation against *E. coli* (ATCC25922). *T. Schimperi* had the highest antibacterial activity at 1000 mg/mL, and with this, it had a DZI: 18.5 ± 0.55 mm diameter of the zone of inhibition compared to the other tested extracts. Following this, the extract–extract combination of *T. Schimperi* and *R. prinoides* had the second highest antibacterial activity (DZI: 14.5 ± 0.55 mm) (Table 7). 

In all the in vitro antibacterial activity evaluations against the *E. coli* (both clinical isolate and reference strain), the antibacterial activities of the extracts were totally lower than ciprofloxacin, the positive control. However, the antibacterial activity of ciprofloxacin against *E. coli* (ATCC25922) was higher than its activity against the MDR *E. coli* clinical isolate. This finding showed that the standard *E. coli* (ATCC25922) is a different strain, and the clinically isolated *E. coli* had acquired a multidrug resistance (Table 7). 

In the in vitro antibacterial activity evaluation of the three extracts and the combination against the patient-isolated MDR *K. pneumoniae* extended-spectrum β-lactamases (*ESBL*), *T*. *schimperi* had a concentration-dependent maximum inhibition compared to the tested extracts at corresponding similar concentrations. However, the antibacterial activity of *T*. *schimperi* was relatively lower compared to ciprofloxacin (DZI: 17.83 ± 0.41 mm). In the *T*. *schimperi and R. prinoides* combination, the antibacterial activity was second with the highest zone of inhibition (DZI: 11.90 ± 0.10 mm) (Table 7). 

Regarding activities against *K. pneumoniae* (ATCC700603), *T. schimperi* showed the highest zone of inhibition (DZI: 14.83 ± 0.40 mm) compared to the extracts and the combination. The antibacterial activity of the *R. prinoides* extract also had a concentration-dependent increase across the concentrations tested and had higher antibacterial activity than the activity against the patient-isolated MDR *K. pneumoniae ESBL* (Table 7). In both MDR patient-isolated *K. pneumoniae* extended-spectrum β-lactamases (*ESBL*) and *K. pneumoniae* (ATCC700603), the antibacterial activity of *J. schimperiana* was similar to the negative control (Table 7). 

### 2.8. Minimum Inhibitory Concentration (MIC)

The findings of the MIC of the three hydromethanolic leave crude extracts are displayed in Table 8. In all the extracts, the concentration ranges for MIC determination were evaluated from 64 mg/mL up to 0.03125 mg/mL. Within the tests, the MIC activity of the extracts against patient-isolated MDR *E. coli* ranged from 4 mg/mL up to 8 mg/mL, whereas against standard *E. coli* (ATCC25922), it ranged from 4 mg/mL up to 32 mg/mL. However, the concentration of the standard drug ciprofloxacin for the MIC tested against all *E. coli* strains ranged from 0.0024 to 5 µg/mL. 

Both *T. schimperi* alone and its combination with *R. prinoides* had the lowest MIC value (4 mg/mL) in the test against the clinical isolate MDR *E. coli*. However, *T. schimperi* was the only extract with the lowest MIC (4 mg/mL) among the extracts evaluated against *E. coli* (ATCC25922). The positive control (ciprofloxacin) had the lowest MIC value compared to all of the independent extracts and the two extracts combination. In the effect against the patient-isolated MDR *E. coli*, ciprofloxacin MIC was identified at 0.156 µg/mL, but the MIC value was lowest enough in *E. coli* (ATCC25922) (0.0048 µg/mL). The lowest MIC of *T. schimperi* in the antibacterial activity evaluation against patient-isolated MDR *K. pneumoniae* was 8 mg/mL but it was 4 mg/mL (MIC) for activity against *K. pneumoniae* (ATCC700603) (Table 8).

### 2.9. Minimum Bactericidal Concentration (MBC)

Among the extracts tested for MBC determination, *T. schimperi* had the lowest MBC (16 mg/mL) in the evaluation against patient-isolated MDR *E. coli*, which was was similar to the same concentration of the MBC against the reference strain *E. coli* (ATCC25922). The standard drug ciprofloxacin had the lowest MBC compared to the extracts. In the test against *E. coli* (ATCC25922), the MBC of ciprofloxacin was 0.0006 µg/mL. The lowest MBC of *T. schimperi* in antibacterial activity evaluation against patient-isolated MDR *K. pneumoniae* was 32 mg/mL but it was 64 mg/mL (MBC) for activity against *K. pneumoniae* (ATCC700603) (Table 8). The ratio of MBC/MIC of the extracts revealed the exact definition of the antibacterial effect. Accordingly, for both *E. coli* strains, *T. schimperi* had a bactericidal effect. *R. prinoides* had a bacteriostatic in effect against patient-isolated MDR *E. coli*. Whereas the *T. schimperi* and *R. prinoides* combination showed bacteriostatic effect in the test against the MDR *E. coli* clinical isolate (Table 8). In this study, the ratio of MBC to MIC of *T. schimperi* alone and in combination with *R. prinoides* in the test against *K. pneumoniae* (ATCC700603) was bacteriostatic. However, in the test against patient-isolated MDR *K. pneumoniae ESBL*, *T. schimperi* showed a bactericidal effect (Table 8). 

### 2.10. Checkerboard Assay

To quantify the interactions, the Fractional Inhibitory Concentration (FIC) index of the checkerboard analysis in this study showed that the interaction of the combinations between *T. schimperi* extract and *ciprofloxacin* (positive control) was categorized as indifferent in the activity against the three bacterial strains except for the MDR *E. coli* clinical isolate (additive). Similarly, according to the FIC index value, the *R. prinoides* and *ciprofloxacin* combinations showed an indifference interaction in the test against all the three bacterial strains except for *E. coli* (ATCC25922) (antagonist) (Table 9). 

### 2.11. Analyses

According to the one-way ANOVA analyses, there was a statistical significance (*p* < 0.05) in the mean differences of each measurement across the column among the three extracts (Table 10). 

#### Correlation between Antioxidant Assays and Phytochemical Contents

The Pearson correlation coefficients between the antioxidant assays and the Total Flavonoids Content (TFC), Total Phenol Content (TPC), and Total Proanthocyanidins Content (TPROC) were analyzed and presented in Table 11. The Total Phenol Contents TPC showed almost a perfect negative correlation with the antioxidant activity value from the DPPH (IC 50%) assay (r = −0.999) at a level of statistical significance (*p* = 0.023). Further, the lowest IC50% means the highest antioxidant activity. Additionally, these analyses revealed that the total flavonoid contents of the extracts correlated significantly with the ABTS assay (mean of scavenging activity; r = 0.999; *p* = 0.032). However, the Total Proanthocyanidins Content (TPROC) did not correlate with the antioxidant activities of the extracts (Table 11). 

## 3. Discussion

The phytochemical screening test results of this study revealed the three extracts contain different phytochemical contents. Primarily, the extracts were evaluated to assess the antibacterial activity against patient-isolated MDR-Uropathogenic bacteria. Importantly, similar to the content of the extracts, several study reports have revealed that medicinal plants containing saponins, phenolics, flavonoids, alkaloids, and tannins have antimicrobial potential as bactericidal or bacteriostatic agents against microbial pathogens [20,21,22].

In the GC-MS analyses of this study, a total of 14 compounds were identified and predicted to be in the hydromethanolic leaf extract of *T. schimperi*. These identified compounds possess many biological properties. Hexanedioic acid, bis (2-ethylhexyl) ester (RT: 19.835; 73.88%) was the main component among the total compounds eluted. Several study reports showed that hexanedioic acid, bis (2-ethylhexyl) ester has numerous biological activities such as antioxidant and antibacterial activities [23,24]. The antioxidant and antibacterial activity of the *T. schimperi* extract in this study is the highest among the tested extracts. Therefore, together with other compounds eluted, hexanedioic acid, bis (2-ethylhexyl) ester offers this important antioxidant and antibacterial activity that is derived from the *T. schimperi* extract. 

Thymol, which is the second major component of the *T. schimperi* extract during the GC-MS analyses, was reported as possessing different biological activities. Accordingly, thymol was stated to have antibacterial activity including against Gram-negative bacteria strains [25,26]. Therefore, the presence of thymol at a higher concentration in the hydromethanolic extract of *T. schimperi* in this study could be the possible explanation for its higher antibacterial and antioxidant activities compared to the other extracts. 

Obtained results showed that the third compound in the GC-MS analyses of *T. schimperi* extract was o-cymen-5-ol. According to [27], o-cymen-5-ol alone or together with zinc shows direct antimicrobial effects, inhibiting oral disease-related processes, and showing synergistic effects against anaerobes. Further, o-cymen-5-ol was reported by [28] to possess antibacterial activity, absorb specific wavelengths of ultraviolet light, inhibit oxidation, and in cosmetics preparations has antiseptic, anti-acne properties, and extends the shelf life of products. A report from [29] conveyed that o-cymen-5-ol has antibacterial activity. *T. schimperi*’s antibacterial and antioxidant activities possibly could be due to the presence of o-cymen-5-ol alone or together with other phytochemical constituents. 

Similar to the finding of the GC-MS analyses of the *T. schimperi* extract, the major constituent of the hydromethanolic crude extract of *R. prinoides* was hexanedioic acid, bis (2-ethylhexyl) ester. As previously mentioned, this compound has reported to have antioxidant and antibacterial activities [23,24]. 

The second abundant bioactive compound known as β-D-Glucopyranoside (methyl) among the total eluted compounds in the GC-MS analyses of *R. prinoides*, was reported to have antibacterial activity [30]. A study report in [31] also described that β-D-Glucopyranoside (methyl), which is a chemical component found in phenols and flavonoids, could potentially have an antimicrobial, antibacterial effect, and could be used as a medication for different diseases. Therefore, the antibacterial effects of the *R. prinoides* in this study might be mediated from the biological activities of these identified compounds existing in adequate amounts during the GC-MS analyses. 

In this study, all the hydromethanolic extracts of the three plant extracts showed antioxidant activity. And *T. schimperi* had the highest DPPH free radical scavenging activity which was seen in *T. schimperi*, followed by the *R. prinoides* extract. Several studies [32,33] have explained that lower IC50 values in a sample indicate higher antioxidant activity. As a result, the observed IC50 value indicated that *T. schimperi* had the lowest IC50 value which indicates the greatest antioxidant activity. Although the *T. schimperi* extract showed the highest IC50 value, it was closer to that of the IC50 of ascorbic acid, showing greater inhibitory effectiveness [34]. For almost all of the extracts, the DPPH assay of this study revealed that the percentage of DPPH inhibition was concentration dependent. This result is similar to the report in [32]. 

The quantitative phytochemical analyses of this study showed that the Total Phenol Content (TPC), Total Flavonoids Content (TFC), and Total Proanthocyanidin Content (TPROC) were found in the highest concentration in the *T. schimperi* extract compared to the other extracts. In the Pearson coefficient analyses, TPC showed a significant negative correlation with DPPH antioxidant activity, which showed that TPCs of the extracts had both hydrogen- and electron-donating abilities of antioxidant capacity. In earlier studies, reports indicated that there is a direct correlation between antioxidant activity and the TPC found in extracts, as phenolic compounds have major antioxidant activity [35,36]. However, current evidence suggests phenolic compounds of plants were reported to have antimicrobial activities [37]. However, factors such as genetic and environmental conditions (like growth season and plant maturity) can cause variations in their values [38].

Also, the Pearson coefficient analyses of this study showed that the highest positive correlation was between total flavonoid content (TFC) and ABTS radical scavenging activity. This study result is in line with previously reported studies [39,40]. These results suggest that this study’s extracts can be used as a natural source of antioxidants. Earlier reports [41,42] stated that flavonoids, which are plant secondary metabolites, demonstrated the ability to scavenge oxygen species (ROS) consisting of free (OH) radicals and further, they are implicated in antibacterial activities [43].

In this evaluation study of hydromethanolic crude extracts, *T. schimperi* had the highest antibacterial activity against the clinical isolates MDR *E. coli* and *K. pneumoniae ESBL* compared to the other extracts. Previously, refs. [44,45] reported that, similar to the current finding, *T. schimperi* oil showed a moderate antibacterial activity on these specific organisms. The finding of this study is similar to several earlier studies [46] which stated that the Ethiopian medicinal plants presented by local healers in the community were reported to have antibacterial activity against MDR bacterial infections. 

The extract–extract combination of *T. schimperi* and *R. prinoides* had similar antibacterial activity in the test against patient-isolated *MDR E. coli* with uncombined *T. schimperi.* However, the antibacterial activity evaluation revealed that the effect against the rest of these three organisms was lower than uncombined *T. schimperi* extract. This result clearly indicates that the combined extracts *of T. schimperi* and *R. prinoides* did not show synergistic activities. This study is different from [47] whose study on *T. schimperi* and *Blepharis cuspidata* combinations showed synergistic activity. This could be due to the difference in the type of combined plant material with *T. schimperi*, or a difference in the extraction method or other undisclosed factors.

Comparatively, the MIC of the *T. schimperi* extract had the lowest MIC compared to the other independent extracts during the antibacterial activity test, except for the MIC (equivalent) of its combination with *R. prinoides* in the test against the clinically isolated MDR *E. coli* and *K. pneumoniae* EBSL. This result further confirmed that *T. schimperi* had the highest antibacterial activities for the entire patient-isolated MDR bacteria and reference strains compared to the tested extracts. This current study finding is in line with [48] which stated that the lower MIC indicates that the plant extracts were stronger in activities of killing or inhibiting the test pathogens and vice versa. Additionally, this study clearly indicated that the ratio of MBC to MIC of *T. schimperi*, except in the test against *K. pneumoniae* (*ATCC 700603*), showed bactericidal activity. 

Among the extracts in this study, *T. schimperi* had the highest zone of inhibition against the MDR microbes tested. This result is in agreement with a study report of [20], which explained that extracts obtained from *C. englerianum* and *E. depauperata* showed a significant zone of inhibition on MDR *E. coli* that ranged from 12 to 25mm in diameter but unlike the current study, the test on *K. pneumoniae* showed a 23–28 mm zone of inhibition. In addition, the present work finds a lower MBC in the test against *K. pneumoniae* than another study on antimicrobial activities of oil extract from the husk of *A. corrorima* and petroleum ether extract of seed of *N. sativa* which were significantly higher than that of the control antibiotic [Gentamycin sulfate, (IZ, 25–30 mm)] [49]. In all of the tests against the strains, the MIC of *T. schimperi* is lower than a study previously reported study, which investigated the MIC value (12.5 mg/mL) for oil from the husk of *A. corrorima* against *Pseudomonas aeruginosa* [49]. The discrepancies in the zone of inhibition and MIC could be potentially due to the type, potency and effectiveness of the medicinal plant, the solvent used for extraction, the bacteria strain, resistant acquisition nature of the bacteria, and also other unseen affecting factors in the laboratory. 

According to the checkerboard analyses of the present study, the interaction of the combinations between the *T. schimperi* and ciprofloxacin combination was additive. This is interpreted as overall antibacterial activity of the extract and the positive control combination equals the sum of the individuals’ effect. Thirdly, following the extract–extract combinations’ activity, the *R. prinoides* extract showed antibacterial activity against the patient-isolated MDR *E. coli* and *K. pneumoniae* ESBL and their reference strains in a concentration-dependent manner, with a slight increased effect in both reference strains. 

## 4. Materials and Methods

### 4.1. Plant Collection and Study Areas

Fresh leaves of *Rhamnus prinoides* were collected from the cultivated garden of a family on 15 May 2021 in Addis Ababa, Ethiopia. Then after, it was identified by a taxonomist at the National Herbarium, Department of Biology, College of Natural and Computational Sciences, Addis Ababa University, and the specimen (number ME 001) was deposited for future reference. The second plant, *Thymus schimperi* leaves (specimen number ME 002), was collected on 30 July 2021 from Debre Birhan town near villages, Amhara Region, Ethiopia which is 120 Km away from Addis Ababa in a northwest direction. The third plant, *Justicia schimperiana* leaves (specimen number MJ 001), was collected from Debre Markos, East Gojjam Zone, Amhara Region, 300 Km away from Addis Ababa, Northwest, Ethiopia. The in vitro antioxidant study was carried out at Addis Ababa University, College of Natural and Computational Sciences, in the Center of Food Science and Nutrition, Addis Ababa, Ethiopia. However, the antibacterial study was carried out in the Ethiopian Public Health Institute (EPHI), Traditional and Modern Drugs Studies Directorate, Pharmacology l Laboratory, Addis Ababa, Ethiopia. The GC-MS analysis of the extracts was performed in at JIJE LaboGlass Private Limited Company, Addis Ababa, Ethiopia. 

### 4.2. Chemicals and Reagents

In this study, the chemicals used were 2,2-diphenyl-1-picrylhydrazyl (DPPH) (Sigma-Aldrich, St. Louis, MO, USA), ascorbic acid (vitamin C), 2,2′-azino-di-(3-ethylbenzthiazoline) sulfonic acid (ABTS) solution (Roche Diagnostic GmbH, Mannheim, Germany), (±)-6-Hydroxy-2,5,7,8-tetramethylchromane-2-carboxylic acid (Trolox^®^) (Sigma-Aldrich, USA), ferrous sulfate, potassium persulfate (Carl Roth GmbH+ Co.KG, Karlsruhe, Germany), ciprofloxacin powder, catechin, gallic acid, Dimethyl sulfoxide (DMSO), and methanol (Alpha Chemika, Maharashtra, India). The solvent methanol was of analytical grade.

### 4.3. Study Design

An in vitro experimental study design was used to carry out the phytochemical screening test and in vitro antibacterial activity evaluation of the selected medicinal plants against the patient-isolated multidrug resistant uropathogenic bacteria. All experimental tests were performed in triplicates alongside the positive and negative controls. 

### 4.4. Plant Extraction

Although a standardized extraction protocol has not been developed for herbal extracts, 20–95% of the solvents substances are frequently used by the herbal medicine industry to prepare plant crude extracts [50]. All three medicinal plants, *Rhamnus prinoides*, *Thymus schimperi*, *and Justicia schimperiana,* were extracted with 80% methanol (and 20% distilled water). Two hundred grams (*Thymus schimperi*), 150 g (*Rhamnus prinoides*), and 250 g (*Justicia schimperiana*) of air-dried powdered plant of materials were placed in a flat-bottom flask filled with 800 mL of methanol and 200 mL (distilled water) of extracting solvents and macerated for 72 h over a rotary shaker at 121 rpm. The suspension was filtered every 24 h with Whatman Number 1 paper (Whatman Ltd. International, Maidstone, UK). The residue was re-macerated for the second and third times with fresh solvent. The resulting filtrate was then concentrated under reduced pressure in a rotary evaporator (R-300) (BUCHI, Flawil, Switzerland). The filtrate residue was further dried, followed by a water bath at 45 °C, until the solvent was removed. After the solvent was evaporated, the remaining crude extracts were diluted with 10 mL of sterile distilled water and kept in an airtight bottle in the refrigerator until the experiment was carried out. 

### 4.5. Phytochemical Screening

The qualitative phytochemical tests for the identification in the hydromethanolic (80% methanol) crude extract of leaves of *Rhamnus prinoides*, *Thymus schimperi*, and *Justicia schimperiana* were carried out by the previously described methods in [51,52,53,54,55,56,57]. All the extracts (0.05 g/mL) were subjected to phytochemical screening which was performed following the standard protocols using different reagents and chemicals for the detection of the following constituents. 

Test for alkaloids: To the filtrate in the test tube, 1 mL of Mayer’s reagent was added drop by drop. The formation of a greenish colored or cream precipitate indicated the presence of alkaloids. 

Test for steroid: Crude extract was mixed with 2 mL of chloroform and concentrated H_2_SO_4_ was added sidewise. A red color produced in the lower chloroform layer indicated the presence of steroids.

Test for flavonoids: Two mL of each extract was added with 2 mL of 2.0% sodium hydroxide, and the formation of an intense yellow color was observed. To this, 2 drops of 70% dilute hydrochloric acid were added and the yellow color disappeared. Formation and disappearance of a yellow color indicates the presence of flavonoids in the sample extract. 

Test for saponins: Crude extract was mixed with 5 mL of distilled water in a test tube, and it was shaken vigorously. The formation of stable foam was taken as an indication for the presence of saponins.

Test for anthocyanin: Approximately 2 mL of the prepared plant extracts were added to 2 mL of 2N HCl and ammonia. The appearance of a pink red coloration that turned blue violet indicated the presence of anthocyanin.

Test for coumarin: About 3 mL of 10% NaOH was added to 2 mL of plant extracts. The formation of a yellow color was an indication for the presence of coumarins.

Test for phenols and tannins: Two milliliters of 5% solution of FeCl_3_ was added to 1 mL crude extracts. A black or blue green color indicated the presence of tannins and phenols. 

Test for terpenoids: Two milliliters of chloroform was added to the 5 mL of plant extract and evaporated on the water path and then boiled with 3 mL of H_2_SO_4_ concentrated. A grey color formed indicated the entity of terpenoids. 

### 4.6. Inoculum Preparations and Standardization

The patient-isolated MDR bacterial strains from the urine sample came from the urine culture, *E. coli*, and *K. pneumoniae ESBL* and the reference strains such as *E. coli* (*ATCC25922*), and *K. pneumoniae* (*ATCC700603*) were utilized to evaluate the antibacterial activities of crude plant extracts. The clinical isolates were obtained from Arsho Medical Laboratory, Addis Ababa, Ethiopia. Each bacterial strain was inoculated and incubated (Incubator Memmert, Schwabach, Germany) for 24 h at 37 °C. To prepare the final inoculum, the cultures were diluted with fresh Mueller–Hinton broth to achieve the required standardized turbidity of bacterial suspension (Optical Densities, OD) by measuring using a UV–Visible Spectrophotometer (Thermo Scientific Evolution 60S, Madison, WI, USA) at 625 nm, (OD values range from 0.08 to 0.1). It was equivalently matched with the turbidity of the 0.5 McFarland barium sulfate standard corresponding to 1.0 × 10^8^ colony forming units (cfu/mL) following the guidelines of the Clinical and Laboratory Standard Institute [58]. The turbidity of the inoculum tube was adjusted visually by either adding bacterial colonies or by adding sterile normal saline solution to that of the already prepared 0.5 McFarland standard. Finally, the inoculum amount of the bacteria was 5.0 × 10^5^. 

### 4.7. Antibacterial Assay of Plant Extracts

Antibacterial activity of hydromethanolic leaf extracts was determined by the agar well diffusion method [59,60]. Multidrug-resistant (MDR) bacterial colonies in a subculture on blood agar plate media were incubated for 24 h at 35 ± 2 °C. The MDR bacterial colonies were dissolved in a normal saline solution with a turbidity equivalent to the 0.5 McFarland standard. One hundred μL of each MDR bacterium was inoculated in Mueller–Hilton agar (MHA) by spreading the bacterium on the surface of the agar using a sterilized glass spreader. After thirty minutes of inoculation, the wells were prepared using a sterilized steel cork borer (1 cm in diameter). The labeled four wells were with 100 μL of 250, 500, 750, and 1000 mg/mL of the crude extracts, resulting in the final concentration of 25, 50, 75, and 100 mg/well, respectively. All plates were then incubated aerobically at 35 ± 2 °C for 24 h, and then, the zone of inhibition was measured using a ruler. The experiment was carried out in three independent tests for each bacterial strain and the mean of the zones of inhibitions was calculated for each extract. A 4% DMSO was used as a negative control. Ciprofloxacin (5 μg) was applied as a positive control. 

### 4.8. Minimum Inhibitory Concentration

The minimum inhibitory concentrations (MICs) for all the crude extracts were evaluated against *E. coli* and *K. pneumoniae ESBL* and their reference strains were determined in triplicate using the 96-wells method in Mueller–Hinton broth according to CLSI (Clinical Laboratory Standardization Institute) [61]. A 4% DMSO was used to dilute crude plant extracts. To determine the MICs of each of the extracts, the concentrations prepared for each of the extracts, ranged between 0.03125 mg/mL and 64 mg/mL, while ciprofloxacin concentration ranged between 0.0024 and 5 μg/mL. All the tested extracts and ciprofloxacin concentrations were prepared by serial dilution in double-strength MHB. An amount of 100 µL of each of the bacterial strains was inoculated to each well. Blank Mueller–Hinton broth was used as a negative control. The wells were inoculated with the standardized (0.5 McFarland standard) bacterial inoculum and incubated at 37 °C for 24 h. The MIC was defined as the lowest concentration that showed no growth in the Mueller–Hinton broth. The result of bacterial inhibition was judged by comparison with growth in the positive and negative controls [62].

### 4.9. Minimum Bactericidal Concentration (MBC)

MBC was determined by a method described in different studies [63]. In this method, the contents of all wells containing a concentration of the crude extracts above the minimum inhibitory concentration (MIC) value from each triplicate, in the MIC determination test, was streaked on Mueller–Hinton agar with a wire loop aseptically cleaned and incubated at 37 °C for 24 h. The lowest concentration of the extract which showed no bacterial growth after incubation was observed for each triplicate and noted as the MBC. The average value was taken for the MBC of test material against each bacterium. Further, the ratio of MBC/MIC of the extracts indicated the exact definition of the antibacterial effect. If the ratio MBC/MIC was ≤4, the effect was considered as bactericidal but if the ratio MBC/MIC was >4, the effect was defined as bacteriostatic [64]. 

### 4.10. Checkerboard Assay

Checkerboard analysis was used to determine the impact on potency of the combination of the extract and the antibiotic in comparison to their individual activities. This comparison is then represented as the Fractional Inhibitory Concentration (FIC) index value. The FIC index value takes into account the combinations that produce the greatest change from the individual antibiotic’s MIC. To quantify the interactions between the extracts and the antibiotic being tested (the FIC index), the following equation is used: FIC index = (MIC of extract in combination/MIC of extract alone) + (MIC of antibiotics in combination/MIC of antibiotics alone). The combination was considered synergistic for ∑FIC ≤ 0.5, additive for 0.5 < ∑FIC ≤ 1, indifferent for 1 < ∑FIC ≤ 4, and antagonistic for ∑FIC > 4, according to the EUCAST definition [65]. 

### 4.11. In Vitro Antioxidant Studies

In these in vitro antioxidant assays, 1 g of each of the extracts was dissolved in 20 mL of 99% methanol to make a concentration of 50 mg/mL and then diluted to prepare the series concentrations for antioxidant assays. For comparison in all the assays, reference chemicals were used.

### 4.12. DPPH Radical Scavenging Assay

Free radical scavenging ability of the extracts was tested by the 2,2-diphenyl-1-picrylhydrazyl (DPPH) radical scavenging assay as described by [66,67]. The hydrogen atom-donating ability of the plant extracts was determined by the de-colorization of the methanol solution of 2,2-diphenyl-1-picrylhydrazyl (DPPH). DPPH produces a violet/purple color in methanol solution and fades to shades of a yellow color in the presence of antioxidants. A solution of 0.004% of DPPH (4 mg/100 mL) in methanol was prepared, and 3 mL of this solution was mixed with 1 mL of the extract in methanol at different concentrations (0.02–0.2 mg/mL). The reaction mixture was vortexed thoroughly and left in the dark at room temperature for 30 min. Ascorbic acid (3 mg/10 mL) was used as a reference standard while a methanol solution of DPPH was used as a control. The optical density of the mixture was measured at 517 nm using (Lambda 950 UV/VIS NIR spectrometer (PerkinElmer, Llantrisant, UK)). The percentage of the DPPH radical scavenging activity was calculated using the following equation: percentage of DPPH radical scavenging activity = {(A0 − A1)/A0} × 100; where A_0_ is the absorbance of the control, and A_1_ is the absorbance of the extract. The IC50 value was determined by using the linear regression equation Y = Mx + C; where, Y = 50, and M and C values were derived from the linear graph trendline (for both the standard and samples). The experiment was repeated three times at each concentration. 

### 4.13. ABTS Radical Scavenging Assay

For the ABTS assay, the procedure followed the method of [68] with some modifications. The stock solutions included 7.4 mM ABTS^+^ solution and 2.6 mM potassium persulfate solution. The working solution was then prepared by mixing the two stock solutions in equal quantities and allowing them to react for 24 h at room temperature in the dark. The solution was then diluted by mixing 1 mL ABTS^+^ solution with 60 mL methanol to obtain an absorbance of 1.1 ± 0.02 units at 734 nm using PerkinElmer (Lambda 950 UV/VIS NIR spectrometer). Fresh ABTS^+^ solution was prepared for each assay. The extracts (40 µL) were allowed to react with 2850 µL of the ABTS+ solution for 2 h in a dark condition. Then, the optical density was taken at 734 nm using the spectrophotometer. The standard curve was linear at 300 µM Trolox. Results were expressed in mM Trolox equivalents (TE)/g of the extract. Additional dilution was needed if the ABTS value measured was over the linear range of the standard curve. The ABTS scavenging capacity of the extract was compared with that of the Trolox standard. All determinations were performed in triplicate (*n* = 3). 

### 4.14. Ferric Reducing Antioxidant Power (FRAP) Assay

The total antioxidant potential of the crude extracts was determined according to a method described by [69] as a measure of antioxidant power. The assay was based on the reducing power of a compound (an antioxidant). A potential antioxidant reduced the ferric ion (Fe^3+^) to the ferrous ion (Fe^2+^); the latter forms a blue complex (Fe^2+^/tripyridyltriazine (TPTZ)). The FRAP reagent consisted of 300 mM acetate buffer (pH 3.6), 10 mM TPTZ in 40 mM HCl, and 20 mM FeCl_3_∙6H_2_O. The FRAP reagent was prepared by mixing the acetate buffer, TPTZ solution, and FeCl_3_∙6H_2_O solution in a proportion of 10:1:1 (*v*/*v*/*v*). Briefly, an aliquot of an appropriately diluted sample (1 mL) was mixed with 3 mL of freshly prepared FRAP reagent and mixed thoroughly. The reaction mixture was incubated at 37 °C for 30 min. The optical density of the mixture was measured at 593 nm versus blank using PerkinElmer (Lambda 950 UV/VIS NIR spectrometer). Ferrous sulphate (0–1000 mM) was used to plot a calibration curve for quantification, and the results were expressed as mmol Fe^2+^ per 100 g of the extract. All samples were analyzed in triplicate. 

### 4.15. Determination of Total Phenols

The total phenolic content of the three extracts was determined using the modified Folin–Ciocalteu method [70]. An amount of 0.0625 g of gallic acid/standard chemical was dissolved in 50 mL of methanol. Here, the extracts (50 μL of the 50 mg/mL (stock solution) of each) were mixed with 5 mL of Folin–Ciocalteu reagent (previously diluted with distilled water 1:10 *v*/*v*) and 4 mL (75 g/L or 7.5%) of sodium carbonate (initially, it was kept in the refrigerator for the last 24 h). The mixture was vortexed for 15 s and allowed to stand for 30 min at 37 °C for the color to develop. The optical density was measured in triplicate at 765 nm using PerkinElmer (Lambda 950 UV/VIS NIR spectrometer) AJI-C03 UV-VIS spectrophotometer (PerkinElmer, Llantrisant, UK). 

### 4.16. Determination of Total Flavonoids

Total flavonoids were estimated using the method of [71]. In this study, the standard chemical quercetin reagent (0.0125 g) was dissolved in 50 mL of methanol and run at a dose of 25–225 μL. One mL of 2% AlCl_3_6H_2_O which was dissolved in 100 mL of water in a volumetric flask was added to each of the extracts and to the standard. Then, it was added to 50 μL of *R. prinoides* and *J. schimperiana*, and to 150 μL of *T. schimperi* and finally allowed to stand for 60 min at room temperature before the optical density was measured at 415 nm using an UV-VIS spectrophotometer. 

### 4.17. Quantitation of Total Proanthocyanidins

The total proanthocyanidin was measured by the vanillin/HCl method according to [72]. Briefly, 500 μL (0.5 mL) of *T.* schimperi and *J. schimperiana*, and 100 μL (0.1 mL) of *R. prinoide* from a 50 mg/mL stock solution was added to 3 mL of a 4% vanillin–methanol solution and hydrochloric acid (1.5 mL). The mixture was left for 15 min at room temperature, and then, the optical density was read at 500 nm. The Total Proanthocyanidins Content was calculated from the standard curve for catechin. 

### 4.18. Gas Chromatography-Mass Spectrometry (GC-MS) Analysis

To identify and characterize the bioactive chemicals’ profile of the hydromethanolic leaf crude extracts, a 1 μL amount of concentration from each of the extracts of the *T. schimperi*, *R. prinoides*, and *J. schimperiana* extracts (of 50 mg/mL stock solution) was injected into a gas chromatography system (Agilent Technologies 7890B, Santa Clara, CA, USA) coupled with an inert mass spectrometer (Agilent 5977B) with Single Quadrupole. The separation of the crude extract was achieved using a DB-5ms, capillary column (30 m × 0.25 mm × 0.25 μm) via an inlet split/splitless mode. Helium was used as the carrier gas with a linear velocity/column flow of 1 mL/min. The injector temperature was set at 250 °C, and the oven temperature was kept at 110 °C for 2 min, and then increased by 10 °C/min rate to 200 °C and then by 5 °C/min rate to 280 °C for 9 min. Further, the mass spectrometer experimental condition was explained as follows: Ionization mode: EI; EMV mode: Gain Factor; Gain Factor: 1; Transfer line temperature: 280 °C; Ion Source temp: 230 °C; Quad temp: 150 °C; Solvent delay: 3 min; and Acquisition mode: SCAN. Finally, the NIST 14 mass spectrometry database library was used to identify the bioactive compounds. 

### 4.19. Data Analysis

In the antioxidant study, data were entered and statistically analyzed using SPSS Version 25 (SPSS Inc., Chicago, IL, USA) and Microsoft Office Excel 2017 (Microsoft, Redmond, WA, USA). All measurements were performed in triplicate, and results were expressed as mean ± standard deviation (SD). A one-way ANOVA was performed to compare the mean differences of the phytochemical contents (total flavonoids, phenol, and proanthocyanidin contents) among the three extracts. If any mean difference exists in the phytochemical contents, the List Significant Difference (LSD) test was used to identify where the differences were. The Pearson correlation coefficient (r) was used to analyze the association among total flavonoids, phenol, and proanthocyanidin contents versus the antioxidant activity (DPPH [IC 50%], ABTS [mean] and FRAP [mean]). In all the statistical tests, *p* ≤ 0.05 was considered as statistically significant. The findings of the antibacterial effects of the crude extracts in terms of zone of inhibitions, MIC, and MBC were summarized in the form of means (± standard deviations). The presence of phytochemical screening results was classified as highly present (+++), moderately (++), slightly (+) and absent (–), respectively. Finally, to identify and characterize the bioactive chemicals profile of the crude extracts, GC-MS analysis was used. 

## 5. Conclusions

The study found that the *Thymus schimperi* extract demonstrated the highest antioxidant and antibacterial activity against MDR *E. coli* and *K. pneumoniae ESBL* and the reference strains. The interaction with ciprofloxacin was classified as additive in *E. coli* MDR. *Thymus schimperi* extract had the highest total phenol, flavonoid, and proanthocyanidin contents, followed by the *Rhamnus prinoides* extract. GC-MS analyses predicted 14 compounds in the *Thymus schimperi* extract, 6 compounds in the *Rhamnus prinoides*, and 5 compounds in *Justicia schimperiana*, which may explain the best antibacterial and antioxidant activity against these strains. Additionally, these medicinal plants could be used as a natural source of antioxidants.

## 6. Recommendation

This study should be expanded using in vivo assays against the targeted MDR-Uropathogenic-induced infections. Similar to the current study and based on multiple study reports, the identified bioactive compounds that are found in the hydromethanolic crude plant extracts with antibacterial activity against MDR-Uropathogenic bacteria should be tested in separate compounds or in combination with each other, or combined with other known compounds with antibacterial activity using both in vitro and in vivo models for antibacterial evaluation. 

## Figures and Tables

**Table 1:** Preliminary phytochemical evaluation of the three hydromethanolic crude leaf extracts.

SN.	Phytochemical Constituents	*T. schimperi*	*R. prinoides*	*J. schimperiana*
1	Saponins	++	+	+++
2	Steroids	-	-	-
3	Alkaloids	++	++	-
4	Anthocyanin	-	+	-
5	Coumarins	+	+	++
6	Flavonoids	++	++	++
7	Terpenoids	-	-	-
8	Phenol	+++	+++	+++
9	Tannin	+++	+++	+++

+: Presence; -: absence; (+): low; (++): moderate; and (+++): highest.

**Table 2:** Quantity (Mean ± SD) of phytochemicals of the three hydromethanolic crude extracts.

Sample Type	Flavonoids (mg Q˟ E/100 g)	Phenol (mg GA^†^ E/100 g)	Proanthocyanidins (mg C^‡^E/100 g)
*T. schimperi*	227.96 ± 3.69	274.50 ± 5.29	1618.06 ± 1.32
*R. prinoides*	90.39 ± 0.35	229.21 ± 2.82	406.24 ± 7.58
*J. schimperiana*	82.43 ± 2.53	83.59 ± 2.19	1037.18 ± 4.06

The standard chemicals: Q˟: quercetin; GA^†^: gallic acid; and C^‡^: catechin.

**Table 3:** The bioactive compounds identified through GC-MS analyses from the hydromethanolic *T. schimperi* crude extract.

Peak #	Name of the Compound (CPD)	Formula	MW	RT	Nature (CPD)	%Area
1	α-Terpineol	C_10_H_18_O	154.25	4.67	Menthane monoterpenoids	0.208649
2	Thymoquinone	C_10_H_12_O_2_	164.201	5.31	Monoterpene	0.152454
3	Thymol	C_10_H_14_O	150.22	5.80	Monoterpenoid phenol	11.67551
4	o-Cymen-5-ol	C_10_H_14_O	150.221	5.93	Phenolic compound	7.944895
5	Rosefuran	C_10_H_14_O	150.22	6.29	Aromatic	0.313204
6	p-tert-Butylcatechol	C_10_H_14_O_2_	166.217	7.85	Catechol derivative organic (CPD)	2.194801
7	p-Cymene-2,5-diol	C_10_H_14_O_2_	166.2170	9.60	Essential oil	0.192658
8	4H-1,3,2-Dioxaborin, 6-ethenyl-2-ethyl-4-methyl-4-(2-methylpropyl)-	C_12_H_21_BO_2_	208.11	10.41	-	0.167239
9	Phthalic acid, 2-cyclohexylethyl butyl ester	C_20_H_28_O_4_	332.4	11.71	Ester	0.14886
10	Hexadecanoic acid, methyl ester	C_17_H_34_O_2_	270.4507	13.39	Ester	0.545681
11	E-10-Methyl-11-tetradecen-1-ol propionate	C_18_H_34_O_2_	268.24	15.65	Fatty alcohol esters	0.254021
12	Linolenic acid, methyl ester	C_19_H_32_O_2_	292.5	15.73	Esterified form of linoleic acid	1.434284
13	Phytol, acetate	C_22_H_42_O_2_	338.5677	15.87	Diterpenoid	0.883479
14	Hexanedioic acid, bis(2-ethylhexyl) ester	C_22_H_42_O_4_	370.5665	19.84	Diester of 2-ethylhexanol and adipic acid	73.88426

MW: molecular weight; RT: retention time.

**Table 4:** The bioactive compounds identified through GC-MS analysis from the hydromethanolic crude *R. prinoides* extract.

Peak #	Name of the Compound (CPD)	Formula	MW	RT	% Area	Nature (CPD)
1	Undecanoic acid	C_11_H_22_O_2_	186.29	8.008	1.42	Fatty acid
2	beta-D-Glucopyranoside, methyl	C_7_H_14_O_6_	194.1825	9.719	10.03	O-glycosyl
3	Desulphosinigrin	C_10_H_17_NO_6_S	279.31	11.132	8.28	Carbohydrate
4	δ-Thionodecalactone	C_10_H_18_OS	186.314	15.859	0.51	Lactone
5	Phenol, 3-phenoxy-	C_12_H_10_O_2_	186.21	19.172	0.41	Phenol
6	Hexanedioic acid, bis(2-ethylhexyl) ester	C_22_H_42_O_4_	370.6	19.755	79.36	Diester of 2-ethylhexanol and adipic acid

MW: molecular weight; RT: retention time.

**Table 5:** The bioactive compounds identified through GC-MS analysis from the hydromethanolic crude *J. schimperiana* extract.

Peak #	Name of Compounds	Formula	MWT	RT	% Area	Nature (CPD)
1	4-Hydroxy-9-vinyladamantane-2,6-dione	C_12_H_14_O_3_	206.24	12.288	6.80	-
2	Debrisoquine	C_10_H_13_N_3_	175.23	13.364	12.18	Member of isoquinolines and a carboxamidine
3	1-Methyl-4-[4,5-dihydroxyphenyl]pyridinium bromide	C_12_H_12_BrNO_2_	282.13	14.239	10.62	-
4	Methyl 8,11,14-heptadecatrienoate	C_18_H_30_O_2_	278.43	15.727	10.79	-
5	Hexanedioic acid, mono(2-ethylhexyl)ester	C_14_H_26_O_4_	258.35	19.841	59.60	-

**Table 6:** DPPH inhibition (%) by the three hydromethanolic leave crude extracts.

Tested Samples	% Inhibition of DPPH						
	0.02 mg/mL	0.04 mg/mL	0.08 mg/mL	0.12 mg/mL	0.16 mg/mL	0.2 mg/mL	IC50%
*T. schimperi*	27.86	44.28	78.87	88.64	88.64	89.29	4.49
*R. prinoides*	14.95	37.28	41.87	57.71	75.78	84.03	9.79
*J. schimperiana*	5.12	6.01	16.46	17.93	24.55	35.03	30.68
Ascorbic acid (standard)	34.20	47.14	78.34	90.32	94.94	96.78	3.63

**Table 7:** The in vitro antibacterial activity of the hydromethanolic crude leave extracts against the MDR clinical isolate and standard bacteria strain.

Plant Extracts (Against B_1_)	*E. coli* Clinical Isolate (*MDR*)			
	Zone of Inhibition (mm): Mean ± SD			
	250 mg/mL (Extract)	500 mg/mL	750 mg/mL	1000 mg/mL
*T. schimperi*	17.17 ± 0.41	19.1 ± 0.98	19.70 ± 0.29	20.00 ± 0.00
*R. prinoides*	8.83 ± 0.26	9.5 ± 0.55	10.83 ± 0.41	11.00 ± 0.00
*J. schimperiana*	8.00 ± 0.00	8.00 ± 0.00	8.00 ± 0.00	8.00 ± 0.00
*T. schimperi and R. prinoides*	17.17 ± 0.41	19.17 ± 0.98	19.50 ± 0.49	20.00 ± 0.00
Ciprofloxacin (5 µL/mL)	33.00± 0.00			
Negative control	8.00 ± 0.00	8.00 ± 0.00	8.00 ± 0.00	8.00 ± 0.00
**Plant extracts (Against B_2_)**	***E. coli* (*ATCC25922*)**			
	**Zone of Inhibition (mm): Mean ± SD**			
	250 mg/mL (Extract)	500 mg/mL	750 mg/mL	1000 mg/mL
*T. schimperi*	15.67 ± 0.52	17.67 ± 0.52	18.33 ± 0.52	18.5 ± 0.55
*R. prinoides*	8.83 ± 0.26	10.5 ± 0.55	10.66 ± 0.52	11.5 ± 0.55
*J. schimperiana*	8.00 ± 0.00	8.00 ± 0.00	8.00 ± 0.00	8.00 ± 0.00
*T. schimperi and R. prinoides*	10.17 ± 0.40	11.65 ± 0.35	12.00 ± 0.00	14.5 ± 0.55
Ciprofloxacin (5 µL/mL)	37.00± 0.00			
Negative control	8.00 ± 0.00	8.00 ± 0.00	8.00 ± 0.00	8.00 ± 0.00
**Plant extracts (Against B_3_)**	***MDR K. Pneumoniae CI***			
	**Zone of Inhibition (mm): Mean ± SD**			
	250 mg/mL (Extract)	500 mg/mL	750 mg/mL	1000 mg/mL
*T. schimperi*	12.5 ± 0.55	13.5 ± 0.55	14.00 ± 0.00	14.50 ± 0.55
*R. prinoides*	8.50 ± 0.50	8.75 ± 0.24	9.00 ± 0.00	10.00 ± 0.00
*J. schimperiana*	8.00 ± 0.00	8.00 ± 0.00	8.00 ± 0.00	8.00 ± 0.00
*T. schimperi and R. prinoides*	11.00 ± 0.00	11.58 ± 0.52	11.82 ± 0.12	11.90 ± 0.10
Ciprofloxacin (5 µL/mL)	17.83 ± 0.41			
Negative control	8.00 ± 0.00	8.00 ± 0.00	8.00 ± 0.00	8.00 ± 0.00
**Plant extracts (Against B_4_)**	***K. Pneumoniae* (*ATCC700603*)**			
	**Zone of Inhibition (mm): Mean ± SD**			
	250 mg/mL (Extract)	500 mg/mL	750 mg/mL	1000 mg/mL
*T. schimperi*	12.5 ± 0.55	14.00 ± 0.00	14.00 ± 0.00	14.83 ± 0.40
*R. prinoides*	9.00 ± 0.00	10.00 ± 0.00	10.50 ± 0.55	11.00 ± 0.55
*J. schimperiana*	8.00 ± 0.00	8.00 ± 0.00	8.00 ± 0.00	8.00 ± 0.00
*T. schimperi and R. prinoides*	10.33 ± 0.52	11.5 ± 0.55	11.5 ± 0.55	11.5 ± 0.55
Ciprofloxacin (5 µL/mL)	24.00 ± 0.00			
Negative control	8.00 ± 0.00	8.00 ± 0.00	8.00 ± 0.00	8.00 ± 0.00

B1: *E. coli* (CI) MDR; B2: *E. coli* (ATCC25922); B3: *K. pneumoniae* CI; and B4: *K. Pneumoniae* (ATCC700603).

**Table 8:** The MIC and MBC of the extracts and ciprofloxacin.

Extract and Ciprofloxacin	*MDR E. coli* (*CI*)			*E. coli* (ATCC25922)		
	MIC	MBC	MBC/MIC	MIC	MBC	MBC/MIC
*T. schimperi*	4	16	4	4	16	4
*R. prinoides*	8	64	8	16	>64	ND
*J. schimperiana*	8	>64	ND	32	>64	ND
*T. schimperi and R. prinoides*	4	32	8	16	64	4
*Ciprofloxacillin*	0.156 µg/mL	0.156 µg/mL	1	0.0048 µg/mL	0.0006 µg/mL	0.125
**Extract and ciprofloxacin**	***MDR K. pneumoniae CI* (*ESBL*)**			***K. pneumoniae* (ATCC700603)**		
	**MIC**	**MBC**	**MBC/MIC**	**MIC**	**MBC**	**MBC/MIC**
*T. schimperi*	8	32	4	4	64	16
*R. prinoides*	16	>64	ND	16	>64	ND
*J. schimperiana*	16	>64	ND	16	>64	ND
*T. schimperi and R. prinoides*	8	>64	ND	8	64	8
Ciprofloxacin (µg/mL)	0.156	0.3125	2	0.156	0.156	1

ND: not determined.

**Table 9:** Results of the checkerboard analysis for the interactions between the extracts and ciprofloxacin combination in the activities against the four bacteria strains.

Item	Types of Bacterial Isolates			
	*MDR E. coli CI*	*E. coli ATCC*	*MDR K. pneumoniae CI* (*ESBL*)	*K. pneumoniae ATCC*
TS_A_	2	2	8	8
TS_C_	1	2	16	8
CPR_A_	0.156	0.0192	1.248	0.312
CPR_C_	0.078	0.0096	0.312	0.624
FIC_TS_	0.5	1	2	1
FIC_CPR_	0.5	0.5	0.25	2
FIC _index1_	1	1.5	2.25	3
Category	Additive	Indifference	Indifference	Indifference
RP_A_	8	16	16	16
RP_C_	8	32	16	32
CPR_A2_	0.156	0.0096	0.624	0.312
CPR_C2_	0.156	0.312	0.312	0.312
FIC_RP_	1	2	1	2
FIC_CPR2_	1	32.5	0.5	1
FIC _index2_	2	34.5	1.5	3
Interaction category	Indifference	Antagonist	Indifference	Indifference

TS_A_: *Thymus schimperi* alone; TS_C_: *Thymus schimperi* combination; CPR_A:_ Ciprofloxacin alone; CPR_C:_ Ciprofloxacin combination; FIC_TS_: Fractional Inhibitory Concentration of *Thymus schimperi;* FIC_CPR_: Fractional Inhibitory Concentration index of Ciprofloxacin; FIC_index1_: Fractional Inhibitory Concentration index of *Thymus schimperi* combined with ciprofloxacin; and RP: *Rhamnus prinoides.*

**Table 10:** One-way analysis of variance (ANOVA)**.**

Extracts	TFC (EC1)	TPC (EC1)	TPROC (EC1)	DPPH (IC50%)	ABTS (EC1)	FRAP (EC1)
*T. schimperi*	227.96 ± 3.69 ^A^	274.50 ± 5.29 ^A^	1618.06 ± 1.32 ^A^	4.49 ^A^	27,296.65 ^A^	2521.60 ^A^
*R. prinoides*	90.39 ± 0.35 ^A^	229.21 ± 2.82 ^A^	406.24 ± 7.58 ^A^	9.79 ^A^	1887.62 ^A^	1810.55 ^A^
*J. schimperiana*	82.43 ± 2.53 ^A^	83.59 ± 2.19 ^A^	1037.18 ± 4.06 ^A^	30.68 ^A^	17,825 ^A^	879.28 ^A^
ANOVA	*p* < 0.05	*p* < 0.05	*p* < 0.05	*p* < 0.05	*p* < 0.05	*p* < 0.05

Mean ± SD (n = 3); measurements in each column that have similar letters (^A^) had statistical difference among the extracts at *p* ≤ 0.05 by the Least Significant Difference (LSD) test; IC50%: Inhibitory Concentration; and EC1: Effective Concentration.

**Table 11:** Correlation matrix showing the relationship between antioxidant indices versu*s* TFC, TPC and TPROC of the three extracts.

		TFC (mg Q˟ E/100 g)	TPC (mg GA^†^E/100 g)	TPROC (mg C^‡^E/100 g)	DPPH (IC50%)	ABTS (Mean)	FRAP (Mean)
TFC	Pearson correlation	1	0.718	0.828	−0.692	0.999 *	0.851
	Sig. (2-tailed)		0.490	0.379	0.513	0.032	0.352
	N	3	3	3	3	3	3
TPC	Pearson correlation	0.718	1	0.204	−0.999 *	0.753	0.976
	Sig. (2-tailed)	0.490		0.869	0.023	0.458	0.138
	N	3	3	3	3	3	3
TPROC	Pearson correlation	0.828	0.204	1	−0.168	0.798	0.410
	Sig. (2-tailed)	0.379	0.869		0.893	0.412	0.731
	N	3	3	3	3	3	3
DPPH (IC50%)	Pearson correlation	−0.692	−0.999 *	−0.168	1	−0.728	−0.968
	Sig. (2-tailed)	0.513	0.023	0.893		0.481	0.162
	N	3	3	3	3	3	3
ABTS (mean)	Pearson correlation	0.999 *	0.753	0.798	−0.728	1	0.877
	Sig. (2-tailed)	0.032	0.458	0.412	0.481		0.319
	N	3	3	3	3	3	3
FRAP (Mean)	Pearson correlation	0.851	0.976	0.410	−0.968	0.877	1
	Sig. (2-tailed)	0.352	0.138	0.731	0.162	0.319	
	N	3	3	3	3	3	3

* Correlation is significant at the 0.05 level (2-tailed); colors show repetitions of correlation. The standard chemicals: Q˟: quercetin; GA^†^: gallic acid; and C^‡^: catechin.

## Data Availability

Chromatogram and fragments of common chemicals will be supplemented if it is necessary.

## List of Abbreviations

ABTS: 2, 2′-azino-di-(3-ethylbenzthiazoline sulfonic acid; ATCC: American Type Culture Collection; CLSI: Clinical and Laboratory Standard Institute; DMSO: Dimethyl sulfoxide; DPPH: 2,2-diphenyl-1-picrylhydrazyl; *E. coli*: *Escherichia coli*; EPHI: Ethiopian Public Health Institute; FIC: Fractional Inhibitory Concentration; FRAP: Ferric Reducing Antioxidant Power; GC-MS: Gas-Chromatography coupled with Mass Spectrometry; IC50%: Inhibitory Concentration 50%; *J. schimperiana: Justicia schimperiana; K. pneumoniae ESBL*: *Klebsiella pneumoniae Extended Spectrum Beta*-*lactamase;* MBC: Minimum Bactericidal Concentration; MDR: Multiple Drug Resistance; MHA: Mueller–Hinton Agar; MHB: Mueller–Hinton Broth; MIC: Minimum Inhibitory Concentration; OD: Optical Density; QE: Quercetin Equivalence; *R. prinoides: Rhamnus prinoides; T. schimperi: Thymus schimperi;* TFC: Total Flavonoid Content; TPC: Total Phenol Content; and TPROC: Total Proanthocyanidin Content.
